# Characterization of bacteria expectorated during forced salivation of the *Phlebotomus papatasi*: A neglected component of sand fly infectious inoculums

**DOI:** 10.1371/journal.pntd.0012165

**Published:** 2024-05-21

**Authors:** Naseh Maleki-Ravasan, Seyedeh Maryam Ghafari, Narmin Najafzadeh, Fateh Karimian, Fatemeh Darzi, Roshanak Davoudian, Reza Farshbaf Pourabad, Parviz Parvizi

**Affiliations:** 1 Department of Parasitology, Pasteur Institute of Iran, Tehran, Iran; 2 Department of Plant Protection, Faculty of Agriculture, Ege University, İzmir, Türkiye; Wadsworth Center, UNITED STATES

## Abstract

The infectious inoculum of a sand fly, apart from its metacyclic promastigotes, is composed of factors derived from both the parasite and the vector. Vector-derived factors, including salivary proteins and the gut microbiota, are essential for the establishment and enhancement of infection. However, the type and the number of bacteria egested during salivation is unclear. In the present study, sand flies of *Phlebotomus papatasi* were gathered from three locations in hyperendemic focus of zoonotic cutaneous leishmaniasis (ZCL) in Isfahan Province, Iran. By using the forced salivation assay and targeting the *16S rRNA* barcode gene, egested bacteria were characterized in 99 (44%) out of 224 sand flies. Culture-dependent and culture-independent methods identified the members of *Enterobacter cloacae* and *Spiroplasma* species as dominant taxa, respectively. Ten top genera of *Spiroplasma*, *Ralstonia*, *Acinetobacter*, *Reyranella*, *Undibacterium*, *Bryobacter*, *Corynebacterium*, *Cutibacterium*, *Psychrobacter*, and *Wolbachia* constituted >80% of the saliva microbiome. Phylogenetic analysis displayed the presence of only one bacterial species for the *Spiroplasma*, *Ralstonia*, *Reyranella*, *Bryobacter* and *Wolbachia*, two distinct species for *Cutibacterium*, three for *Undibacterium* and *Psychrobacter*, 16 for *Acinetobacter*, and 27 for *Corynebacterium*, in the saliva. The abundance of microbes in *P*. *papatasi* saliva was determined by incorporating the data on the read counts and the copy number of *16S rRNA* gene, about 9,000 bacterial cells, per sand fly. Both microbiological and metagenomic data indicate that bacteria are constant companions of *Leishmania*, from the intestine of the vector to the vertebrate host. This is the first forced salivation experiment in a sand fly, addressing key questions on infectious bite and competent vectors.

## Author summary

Female sand flies salivate during feeding on vertebrate blood and natural sugars. During salivation, they may release microorganisms associated with the salivary glands and digestive tract, i.e. viruses, bacteria, and other pathogens, into the feeding substrates. However, the type and the number of bacteria egested during salivation is unclear. Here, by sing the forced salivation experiment and targeting the *16S rRNA* barcode gene, egested bacteria were characterized in sand flies. Culture-dependent and culture-independent methods identified the members of *Enterobacter cloacae* and *Spiroplasma* species as dominant taxa, respectively. The abundance of microbes in the saliva of each *P*. *papatasi* sand fly was determined to be around 9,000 bacterial cells. The lack of a vaccine and the failure of treatment in leishmaniasis provide many motivations to better understand the factors cause the inflammatory response. The findings of this study can improve our insight into measuring the effect of vector-derived bacteria on the improvement or deterioration of leishmaniasis.

## Introduction

Leishmaniases, vector-borne and poverty-related infections with a wide clinical spectrum, are regarded a major public health concern in half of the tropical/subtropical countries of the world. [1,2]. By 2019, it was estimated that 0.5–0.9 million new cases of leishmaniasis occurred annually, resulting in 18,700 deaths and 1.6 million disability-adjusted life years (DALYs) lost [3]. Cutaneous and mucocutaneous forms of leishmaniases (CL and MCL) had the greatest DALY increase in the last 20 years [4], and the most CL cases have been detected in eco-epidemiological “hotspot” areas within the Eastern Mediterranean region [5]. In many of these areas, the outbreaks appear in 10-year intervals [6], but this trend is 1.5 times faster in some countries such as Iran [7]. Zoonotic cutaneous leishmaniasis (ZCL), the most predominant and widespread form of leishmaniasis in the country, is caused by *Leishmania major* and chiefly vectored by the sand fly *Phlebotomus papatasi* (Scopoli) (Diptera: Psychodidae) from gerbils to humans [8,9].

The epidemiology of leishmaniases is complicated and mainlly depends on the features of the parasite and sand fly species, the local ecological characteristics of the transmission areas, past and present exposure of human population to the parasite, and human behavior patterns [5,10–13]. Each leishmania species causes distinct clinical symptoms with different degrees of severity in the hosts, ranging from localized skin lesions to the reticuloendothelial system involvement. Sometimes, a parasite manifests a wide range of symptoms [12,14], but the outcome of the disease is ultimately determined by the interactions of the characteristics of the parasite, the biology of the vector, and the immune responses of the host [15,16].

Apart from the metacyclic promastigote of a sand fly, its infectious inoculum is comprised of many relevant factors derived from the parasite and vector [17]. The *Leishmania*-derived factors mainly include proteophosphoglycans and exosomes, which respectively contribute to disease exacerbation by modulating early innate pathways involved in wound response, as well as to the alteration of cell recruitment/behavior patterns. As for vector-derived infection enhancers, both sand fly saliva and gut microbiota play a role. Sand fly salivary proteins are beneficial for the establishment and promotion of infection. It has been shown that bacteria in the intestinal tract of a sand fly are contributing factors to the development of the *Leishmania* parasite inside the midgut and to the enhancement of parasite-derived infection during co-egestion to the vertebrate host [18–21].

Female vector sand flies feed on vertebrate blood and natural sugars such as sap, nectar, fruits, or secretions Homoptera [22]. They salivate during both types of feeding. In the case of sugar meals, saliva is secreted to break down oligosaccharides with α-glucosidase and starch with amylase, as well as to dilute highly concentrated sugar solutions to facilitate ingestion [23]. In contrast, during blood feeding, the sand fly uses the pharmacologically active components of saliva, with anti-hemostatic, anti-inflammatory and immunomodulatory properties that facilitate the blood-meal intake with the least host resistance [24]. Phlebotomines are basically pool feeders or "telmophages", and they suck blood and lymph from small wounds they make on the bite site [25]. During salivation, the vector insects may release microorganisms associated with the salivary glands and digestive tract, i.e. viruses, bacteria, and other pathogens, into the feeding substrates [17,18,26,27].

Studies have recently shown that bacteria in the sand fly’s intestine can affect the establishment and pathogenesis of the *Leishmania* parasite in the vertebrate host by modulating the immune system [18,20]. This behavior can be deduced simply by comparing the needle infection model with the transmission of pathogens through bites [19]. Now, the following questions are raised: what kind of and how many bacteria are loaded during a *P*. *papatasi* sand fly salivation? The current research was designed and conducted to answer these basic questions.

## Methods

### Ethics statement

The ethical considerations of this study were approved by the institutional animal and human ethical committee under national and international standards with the ethical code: IR.PII.REC.1399.027 by Pasteur Institute of Iran.

### Sand fly collection

Live adult sand flies were captured using a car trap from different areas of Isfahan Province during June and July 2020. A car trap is a newly parked light-colored passenger vehicle used to attract sand flies in the vicinity of rodent burrows at sunset. Following landing on the car, the sand flies were collected using a mouth aspirator and flashlight. The gathered flies were transferred to the Entomology Laboratory of the Department of Parasitology at the Pasteur Institute of Iran and kept in metal cages and inside a net, while maintaining humidity and providing with 10% sucrose. After the flies were acclimated to the laboratory conditions (24–27°C, 80% RH, and 14:10 [L:D]), and the transportation stress was gone, they were starved of sugar for 24 hours, prior to the forced salivation experiment.

### Forced salivation experiment

**Fig 1:**
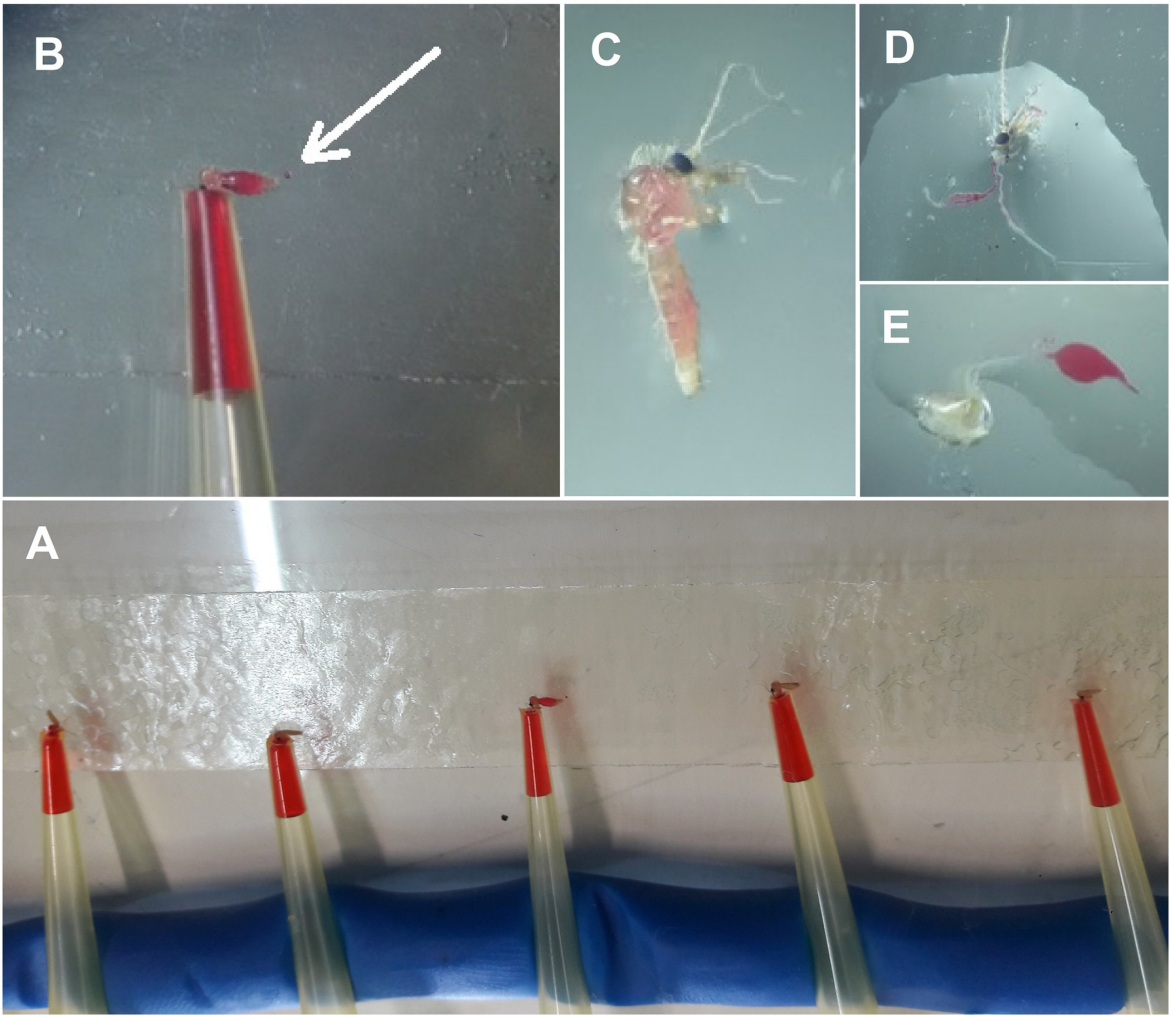
Forced salivation experiment. Sand flies during salivation and feeding (A), showing fully (B) and partially (C) fed specimens with their corresponding dissected guts (D and E). The arrow indicates overfeeding and prediuresis in the sand fly.

This assay is actually the modified method of mosquito vector competence for arboviruses [28]. In brief, the sand flies were taken out from the cage with an aspirator and anesthetized by being placed at -20°C for 2 minutes. To keep anesthesia during the experiment, the flies were transferred to a Petri dish on an ice tray and then examined individually on a clean slide under a stereo microscope. Their wings and legs were cut using tweezers and fine needles, to prevent any movement. As depicted in the Fig 1, the proboscis of each insect was placed in the entrance of a yellow pipette tip with the help of the same tools. Insects were offered sugar meals (10 μl of 10% sucrose solution supplemented with 2% red food colorant) through the yellow pipette tips. The tips, from their wide and narrow sides, were fixed on a glass plate (40 cm × 30 cm) with playdough and a double-sided stick, respectively. After inserting all the proboscises into the tips, the glass plate containing the test specimens was left intact for about one hour in laboratory conditions and away from any air flow, to allow sand flies feeding. Sand flies with red solution in their bodies (complete or partial) were considered as insects with forced salivation (Fig 1A–1E).

### Culture-dependent identification of bacteria egested during forced salivation

Sand flies whose bodies turned red, i.e. those who had salivated and fed, were transferred to a drop of sterile PBS on a glass slide and micro-dissected under a stereo microscope. The dissected guts and the rest of the sugar solution in the yellow pipette tips were separately transferred to falcons containing 5 ml of brain-heart infusion (BHI) broth. The head and the ends of the abdominal segments were mounted on a slide for morphological identification. Each culture medium was named based on the specific code of the corresponding glass slides incubated at 100 rpm at 37°C for 24–48 hours. The opaque media were considered positive specimens and subcultured at above conditions in a BHI agar medium overnight. After obtaining phenotypically unique colonies, pure isolates were photographed. Cultures of pure single colonies were stored in a glycerol containing BHI broth at -80°C, until molecular surveys. In each test period, possible contaminant bacteria of tips, needles, and sand fly cuticles and also sugar meal were cultured separately as negative controls of the experiment.

For molecular identification of bacteria, the genomic DNA of pure isolates was prepared using phenol:chloroform:isoamyl alcohol (24:25:1 v/v) method, in line with a previously described protocol [29]. Five hypervariable (V1–V5) regions of the *16S rRNA* gene of bacteria were targeted to amplify ~800 bp of the gene. The gene amplification and sequencing were carried out based on methods described in the literatures [30,31]. Bacterial identities were determined with focusing on the results of a BLASTn search of *16S rRNA* sequences against nucleotide sequences obtained from the NCBI [32] and leBIBI [33] databases. Sequences obtained through Sanger sequencing were submitted into the GenBank database.

### Culture-independent identification of bacteria egested during forced salivation

Three individual (n = 3) and three pooled (n = 15) specimens were selected from reciprocal gut-saliva collections, from sand flies with successful forced salivation, to characterize bacteria via a culture-independent method. The genomic DNA of specimens was extracted using Sambio TM DNA Extraction Kit (South Korea; Lot: 17F19-16) according to the manufacturer’s protocol, followed by removing RNA contamination via RNase A treatment. Two hypervariable regions, V3-V4, of the bacterial *16S rRNA* gene were amplified by the primers 341F (‘5-CCTAYGGGRBGCASCAG-3’) and 806R (‘5-GGACTACNNGGGTATCTAAT-3’) [34]. Quadruplicate 25-μl amplicons were produced through 35 rounds of amplification involving 5 s at 98°C, 20 s at 56°C, and 20 s at 72°C using Titanium Taq DNA Polymerase (Clontech, Takara, Japan). Then the successful products were purified with QIAquick PCR Purification Kit (Qiagen, Germany). The high-quality sequencing was performed by Beijing Genomics Institute (BGI) in China, using Illumina HiSeq platform. The bioinformatic framework, including metagenomic libraries preparation, filtration of sequencing artifacts, taxonomic assignment of the reads, and other analyses were carried out in accordance with previously reported protocols [35–43].

To determine the phylogenetic position of bacteria found in the sand fly saliva, we compared 404–431 bp of *16S rRNA* fragments of bacteria with the representatives of environmental and pathogenic counterparts validated in List of Prokaryotic names with Standing in Nomenclature (LPSN) [44], using maximum likelihood tree construction method.

### Quantification of bacteria load during sand fly salivation

Four pipette tips, containing sand flies’ saliva, were used to compute the number of bacteria transmitted during fly bites through the smear preparation (n = 2) or optical density (OD) measurement (n = 2). The number of *16S rRNA* gene copies for bacteria with more than 25 reads was also used as a basis for determining the number of bacteria egested into each pipette tip. In this regard, *16S rRNA* gene copy counts for each bacterium identified in this study were first retrieved from the website of the University of Michigan Center for Microbial Systems [45]. Then the number of reads calculated for each bacterium was divided by the average number of *16S rRNA* gene copies determined for that taxon in the above website. Bacteria with unknown identity, specified as “uncultured”, were not included in the calculations.

## Results

### Forced salivation experiment results

A total of 1891 *P*. *papatasi* were caught from Habib-Abad (n = 1003) and Matin-Abad (n = 888) Counties in the hyperendemic focus of ZCL in Isfahan Province, the centr of Iran. A number of 224 female sand flies were examined for forced salivation, which the experiment was successful in 99 (44.20%) sand flies with a range of 21.67%–55.00% (Table 1; Fig 1).

**Table 1:** Details of *Phlebotomus papatasi* sand flies captured from the hyperendemic focus of ZCL Isfahan Province, central of Iran and subjected to forced salivation experiment.

Bacteriological method	Location	Number of sand flies caught (female/male)	Number of sand flies used for forced salivation	Number of sand flies with successful forced salivation (%)
Culture-dependent	Matin-Abad	388 (375/13)	60	13 (21.67)
	Habib-Abad	512 (487/25)	28	12 (42.86)
	Eco-Camp Matin-Abad-1	264 (258/6)	32	22 (68.75)
	Eco-Camp Matin-Abad-2	236 (225/11)	64	30 (46.87)
Culture-independent	Habib-Abad	491 (457/34)	40	22 (55.00) (18 specimens used for NGS, 2 for smear preparation, and 2 for OD measurement)
Total		1891 (1802/89)	224	99 (44.20)

### Culture-dependent identification of bacteria

**Fig 2:**
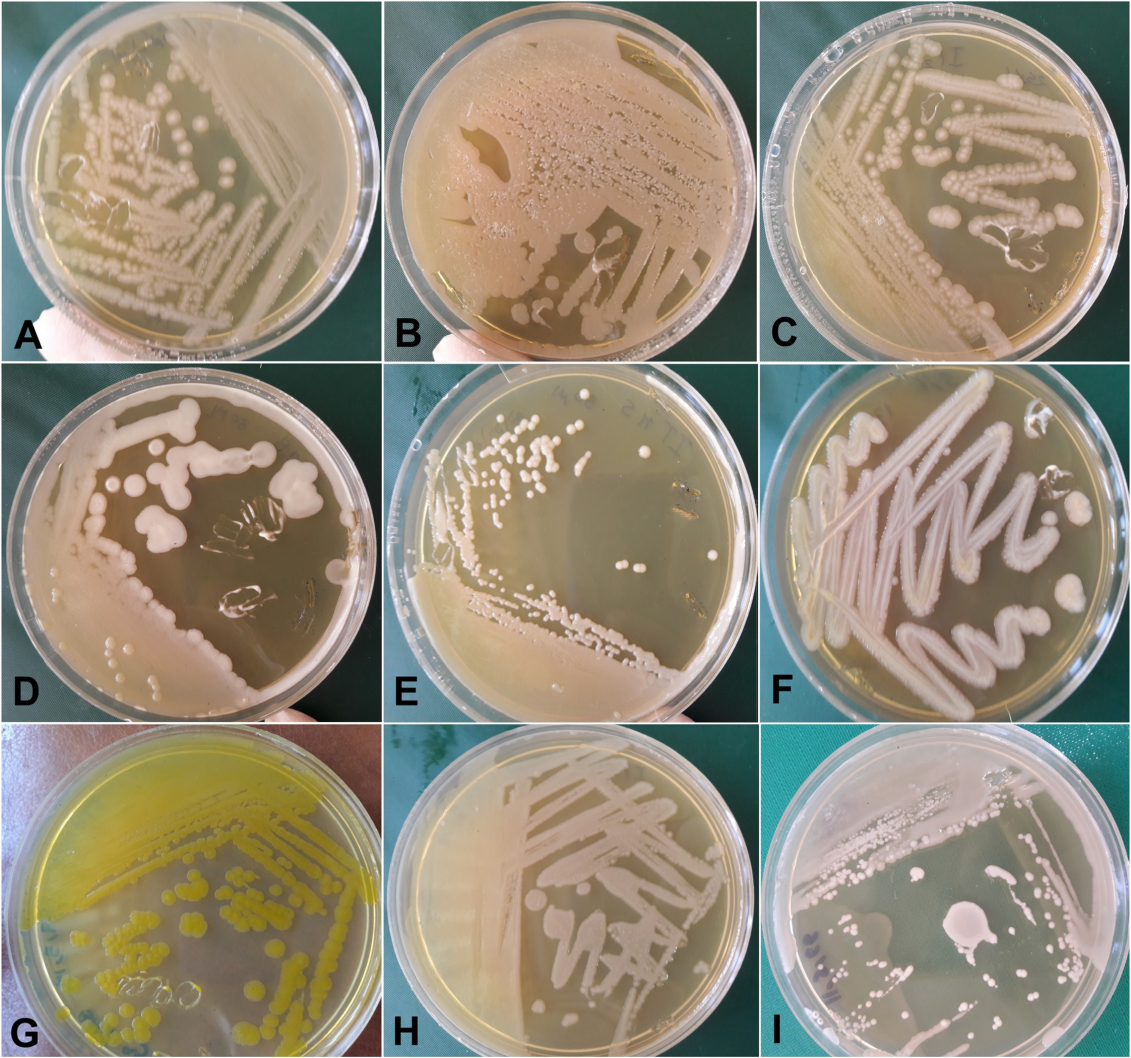
Unique bacterial colonies isolated from sand flies subjected to the forced salivation experiment. A; *Enterobacter hormaechei* (control for cuticle), B; *Priestia flexa* (control for needle), C; *Enterobacter hormaechei* (gut), D; *Priestia aryabhattai* (saliva), E; [*Pseudomonas*] *hibiscicola* (saliva), F; *Providencia rettgeri* (gut), G; *Pantoea stewartii* (gut), H; *Pantoea dispersa* (saliva) and I; *Staphylococcus warneri* (saliva).

Seventy-seven samples from saliva-gut mutual set were used for bacterial culture, single colony preparation, DNA extraction, and PCR-sequencing. About 800 bp of the *16S rRNA* gene of bacteria was successfully sequenced, and the consensus data were deposited in the GenBank under the accession numbers ON314273-ON314424. The sequence analysis determined the presence of 8 families, 8 genera, and 14 species of bacteria in the saliva-gut communities of sand flies. *Enterobacter hormaechei*, a component of the *E*. *cloacae* complex, was detected as the most abundant bacterium in the gut (n = 60) and saliva (n = 61) of sand flies. Two bacteria of *Enterobacter hormaechei* and *Priestia flexa* were found in the cuticle and needle controls, respectively (Fig 2, Table 2).

**Table 2:** Bacteria identified in the saliva of the sand fly *P*. *papatasi* during forced salivation experiment using culture-dependent method.

Location	Bacteria species (family)	Gut	Saliva	Negative control				No.
				Syringe needle	Cuticle	Yellow tip	Sugar meal*	
Matin-Abad	*Enterobacter hormaechei* (Enterobacteriaceae)	7	6	---	---	---	---	13
Habib-Abad	*Enterobacter hormaechei* (Enterobacteriaceae)	6	3	---	---	---	---	9
	*Priestia aryabhattai* (Bacillaceae)	0	1	---	---	---	---	1
	*Stenotrophomonas hibiscicola* (Xanthomonadaceae)	1	1	---	---	---	---	2
Eco-Camp Matin-Abad	*Acinetobacter colistiniresistens* (Moraxellaceae)	0	1	---	---	---	---	1
	*Acinetobacter radioresistens* (Moraxellaceae)	0	1	---	---	---	---	1
	*Enterobacter hormaechei* (Enterobacteriaceae)	47	52	---	1	---	---	100
	*Micrococcus luteus* (Micrococcaceae)	0	1	---	---	---	---	1
	*Pantoea ananatis* (Erwiniaceae)	2	1	---	---	---	---	3
	*Pantoea dispersa* (Erwiniaceae)	6	3	---	---	---	---	9
	*Pantoea stewartii* (Erwiniaceae)	4	2	---	---	---	---	6
	*Priestia flexa* (Bacillaceae)	0	0	1	---	---	---	1
	*Priestia megaterium* (Bacillaceae)	0	1	---	---	---	---	1
	*Providencia rettgeri* (Morganellaceae)	1	0	---	---	---	---	1
	*Staphylococcus epidermidis* (Staphylococcaceae)	1	1	---	---	---	---	2
	*Staphylococcus warneri* (Staphylococcaceae)	0	1	---	---	---	---	1
Total		75	75	1	1	0	0	152

* 10% sucrose solution supplemented with 2% red food colorant

### Culture-independent identification of bacteria

**Fig 3:**
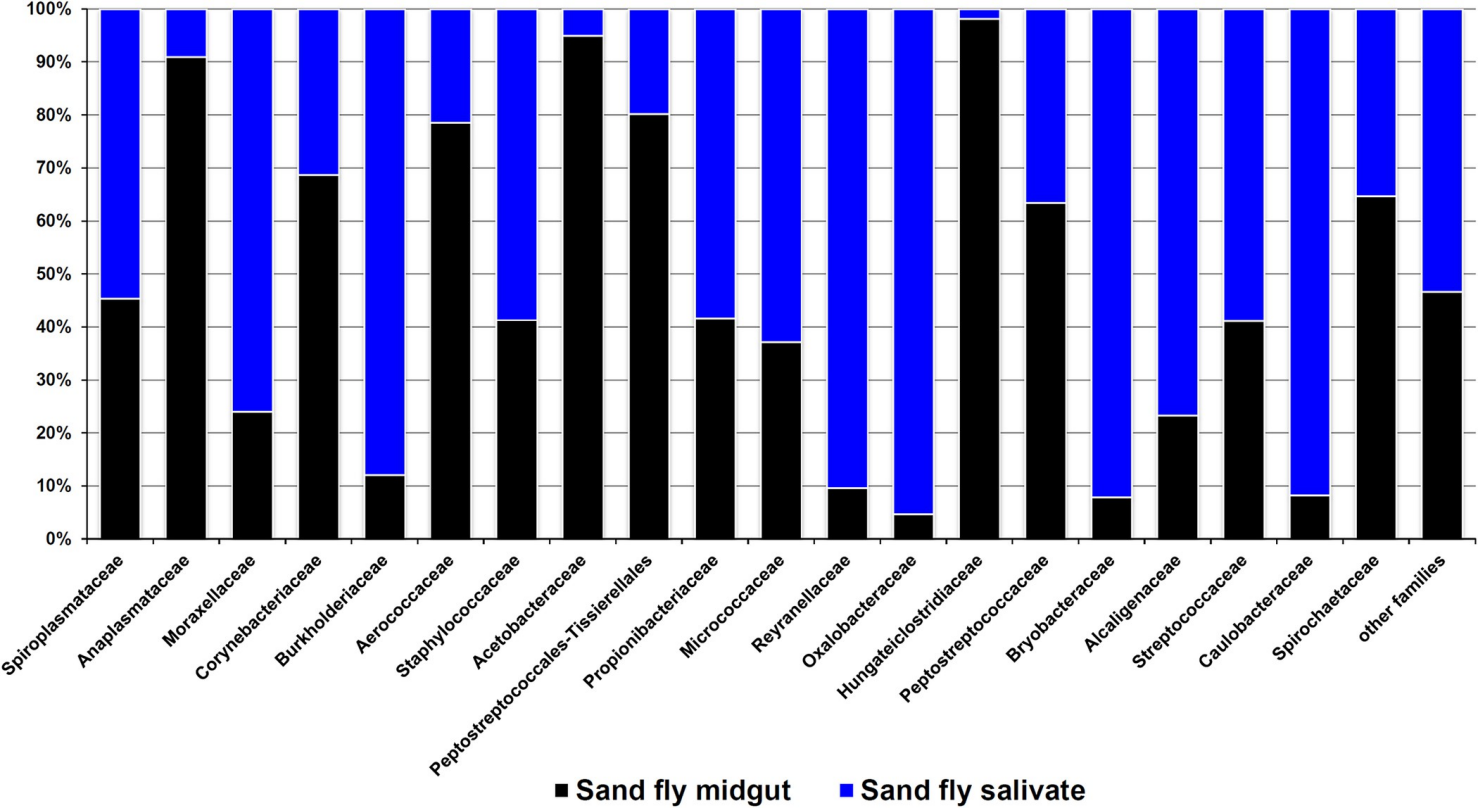
Abundance of bacterial families identified in the saliva and midgut of sand flies using NGS method.

Three individual saliva specimens failed during the quality control (QC) measurement for next-generation sequencing (NGS) analysis. Also, among pooled pairs, only saliva-gut cross pairs were worth analyzing; therefore, our study focused on one of these pairs with successful QC. Illumina HiSeq sequencing platform yielded a total of 122,730 (58154 for gut/64576 for saliva) bacterial *16S rRNA* gene sequences from reciprocal saliva-gut assemblages after trimming, measuring QC, and removing plastid/mitochondrial sequences. The identified operational taxonomic units (OTUs) were divided into 13 phyla, 21 classes, 63 orders, 101 families, 224 genera, and 60 species. The most plentiful families were Spiroplasmataceae, Anaplasmataceae, Moraxellaceae, Corynebacteriaceae, and Burkholderiaceae, respectively (Fig 3). At the genus level, top 10 genera of *Spiroplasma*, *Ralstonia*, *Acinetobacter*, *Reyranella*, *Undibacterium*, *Bryobacter*, *Corynebacterium*, *Cutibacterium*, *Psychrobacter*, and *Wolbachia* accounted for more than 80% of the saliva microbiome. Interestingly, *16S rRNA* gene sequences of some bacteria were found to be more in saliva than intestine (Table 3; Fig 3). The high-throughput sequences of *16S rRNA* gene fragments were deposited in the NCBI database under Sequence Read Archive (SRA) with Bioproject identification number PRJNA861709.

**Table 3:** Bacterial genera found in the saliva and gut of sand flies identified through NGS method.

Feature_ID	No. of sequences		Range of SSU rDNA copy number ($\bar{x}$)*	Feature_ID	No. of sequences		Range of SSU rDNA copy number ($\bar{x}$)	Feature_ID	No. of sequences		Feature_ID	No. of sequences		Feature_ID	No. of sequences		Feature_ID	No. of sequences	
	Gut	Saliva			Gut	Saliva			Gut	Saliva		Gut	Saliva		Gut	Saliva		Gut	Saliva
Spiroplasma	37108	44732	1–2 (1.1)	Bacteroides	203	137	1–7 (3.5)	Globicatella	259	24	Clostridia	7	10	Weeksella	1	4	Rehaibacterium	15	1
Ralstonia	238	1726	2–4 (3.7)	Porphyromonas	177	133	2–5 (3.8)	Ignatzschineria	51	24	uncultured	1	10	Succiniclasticum	1	4	Vibrionimonas	14	1
Acinetobacter	277	1581	1–8 (6.2)	DSSD61	36	132	1–1 (1.0)	Amnipila	38	24	Propioniciclava	9	9	Alcaligenaceae	1	4	Agathobacter	13	1
Reyranella	103	959	1–12 (2.7)	Enhydrobacter	13	130	1–12 (2.7)	Roseburia	27	24	Rhodobacteraceae	1	9	Aerococcaceae	154	3	Candidatus_Alysiosphaera	12	1
Undibacterium	47	914	4–6 (4.7)	Ignavigranum	359	126	3–3 (3)	Helcococcus	237	23	Paenalcaligenes	1	9	Eukaryota	103	3	[Anaerorhabdus]_furcosa_group	11	1
Bryobacter	75	871	2–2 (2.0)	Pedobacter	1	115	2–7 (4.6)	Aerococcaceae	25	23	Christensenellaceae_R-7_group	72	8	Lachnospiraceae	29	3	Coprococcus	11	1
Corynebacterium	1770	739	1–7 (4.3)	Acholeplasma	199	103	2–4 (2.1)	Luteococcus	23	22	Dorea	30	8	UCG-010	8	3	Allofustis	10	1
Uncultured	24	695	---	Bacteroidales	85	103	2–9 (4.7)	Arcobacter	1	22	Lachnospiraceae_NK3A20_group	13	8	Kingella	7	3	Nocardia	9	1
Cutibacterium	466	662	2–6 (3.0)	uncultured	299	102	---	Jeotgalibaca	39	21	Prevotella	8	8	Tessaracoccus	5	3	Modestobacter	9	1
Psychrobacter	444	605	3–6 (4.4)	Lawsonella	63	98	2–2 (2.0)	Solibacillus	1	21	Ochrobactrum	6	8	uncultured	1	3	Rikenellaceae_RC9_gut_group	9	1
Wolbachia	4639	464	1–1 (1.0)	Gemella	7	98	4–4 (4.0)	Flaviflexus	35	20	Petrimonas	1	8	Sphingobacterium	1	3	Erysipelothrix	9	1
Uncultured	17	415	---	JG30-KF-CM45	10	93	2–2 (2.2)	Fastidiosipila	958	19	Anaerococcus	251	7	Gitt-GS-136	1	3	[Eubacterium]_nodatum_group	9	1
Macrococcus	466	399	4–5 (5.0)	Enterobacteriaceae	660	90	1–13 (7.3)	Peptoniphilus	55	19	Alloiococcus	39	7	Firmicutes	1	3	Lachnospiraceae_UCG-010	8	1
Kocuria	83	371	3–3 (3.0)	Bradyrhizobium	90	88	1–2 (1.1)	Filifactor	27	19	Bacillus	26	7	Turicibacter	1	3	Actinomyces	7	1
Jeotgalicoccus	154	355	4–4 (4.0)	Diaphorobacter	21	74	3–4 (3.4)	Dichelobacter	12	19	Family_XIII_AD3011_group	13	7	Aestuariicella	1	3	uncultured	7	1
Oceanibaculum	11	330	1–10 (4.4)	Atopostipes	148	73	4–8 (5.2)	S5-A14a	5	18	Alcaligenaceae	1	7	Erwiniaceae	178	1	UCG-002	7	1
Parapusillimonas	36	326	2–5 (3.0)	Gluconobacter	1319	72	4–5 (4.6)	Yaniella	8	17	Morganella	1	7	Negativicoccus	108	1	Leucobacter	6	1
Fusobacterium	172	324	3–9 (5.3)	Stenotrophomonas	64	62	1–5 (3.6)	Gallicola	12	16	Oceanobacter	1	7	Clostridium_sensu_stricto_1	103	1	Proteiniphilum	6	1
Pseudomonas	91	319	1–9 (4.8)	Glutamicibacter	185	58	4–7 (5.9)	Proteus	1	16	Trueperella	27	6	Micrococcus	61	1	Prevotellaceae_UCG-004	6	1
Bordetella	50	313	3–5 (3.0)	Fusibacter	95	56	1–17 (8.3)	[Eubacterium]_coprostanoligenes_group	48	15	Pigmentiphaga	25	6	Arthrobacter	53	1	Candidatus_Stoquefichus	6	1
Lactococcus	17	304	4–6 (5.6)	Sphingomonas	16	51	1–4 (2.0)	Ezakiella	35	15	Alistipes	5	6	Arcanobacterium	40	1	Acidibacter	6	1
Blastococcus	11	298	3–3 (3.0)	UCG-005	155	48	2–8 (4.3)	Castellaniella	21	15	[Ruminococcus]_gauvreauii_group	1	6	Domibacillus	38	1	Candidatus_Soleaferrea	5	1
Rothia	22	286	3–4 (3.1)	Aerococcus	68	47	3–7 (4.4)	Brachybacterium	19	15	Romboutsia	1	6	Chitinophagaceae	35	1	uncultured	5	1
Treponema	511	280	1–4 (2.2)	Nocardioidaceae	1	45	1–6 (2.3)	Mycoplasma	5	15	Paracoccus	1	6	Nosocomiicoccus	28	1	Anaerovoracaceae	5	1
Obscuribacteraceae*	10	262	1–4 (2.2)	Proteiniclasticum	1	39	1–17 (9.5)	Tissierella	22	14	Lautropia	1	6	Lactobacillus	26	1	Murdochiella	5	1
Staphylococcus	56	241	1–11 (5.7)	Brevundimonas	43	36	1–3 (2.0)	Peptostreptococcus	12	14	Nitrospira	37	5	Enteractinococcus	25	1	Oceanisphaera	5	1
Clostridia_vadinBB60_group	348	232	1–17 (7.9)	Salinicoccus	23	36	4–4 (4.0)	Empedobacter	1	14	Lachnospiraceae_AC2044_group	9	5	TRA3-20	24	1	[Eubacterium]_brachy_group	4	1
Comamonas	103	230	3–11 (6.6)	uncultured	1	36	---	Koukoulia	1	14	Bacteria	8	5	Acidobacteriae	23	1	uncultured	3	1
Lentimicrobium	208	227	2–9 (4.7)	Campylobacter	41	35	1–10 (2.5)	Oligella	80	12	Aliidiomarina	3	5	Planomicrobium	23	1	Muribaculaceae	3	1
Streptococcus	350	220	1–9 (5.3)	W5053	58	34	4–17 (11.9)	Savagea	6	12	KD4-96	1	5	Adhaeribacter	21	1	AD3	3	1
Pantoea	1	190	3–7 (6.9)	Skermanella	1	32	6–7 (6.8)	Dietzia	1	12	Planococcus	1	5	Desulfovibrio	21	1	Blautia	3	1
Myroides	1	184	5–11 (7.7)	Microvirga	1	31	6–7 (6.8)	Cyclobacteriaceae	1	12	Izemoplasmatales	1	5	Lactobacillales	20	1	Syntrophococcus	3	1
Providencia	18	174	7–8 (7.0)	Massilia	1	31	1–9 (6.6)	Paeniclostridium	1	12	Devosia	1	5	Fermentimonas	18	1	Ruminococcaceae	3	1
Flavobacterium	1	170	1–13 (6.6)	Peptostreptococcales-Tissierellales	48	30	2–6 (3.6)	Escherichia-Shigella	31	11	Cellvibrio	1	5	Terribacillus	18	1	Sedimentibacter	3	1
Facklamia	633	166	3–7 (4.5)	Peptostreptococcales-Tissierellales	31	27	2–6 (3.6)	NK4A214_group	8	11	Clostridia_UCG-014	261	4	Exiguobacterium	17	1	Pelagibacterium	3	1
Wohlfahrtiimonadaceae	1	166	1–21 (6.6)	Aerosphaera	1	27	1–12 (5.3)	Brevibacterium	3	11	Dialister	14	4	Monoglobus	16	1			
Peptostreptococcaceae	297	161	4–17 (11.9)	Comamonadaceae	5	26	1–11 (3.3)	uncultured	1	11	uncultured	6	4	Dysgonomonadaceae	15	1			
Peptostreptococcaceae	244	142	4–17 (11.9)	---	---	---	---	Terrisporobacter	42	10	Sporichthyaceae	1	4	Parabacteroides	15	1			

*16S rRNA gene copy counts for bacteria with >25 reads was retrieved from the website of the University of Michigan Center for Microbial Systems.

After ratifying the transmission of microbiota during salivation, the phylogenetic position of 10 top genera, including *Spiroplasma*, *Ralstonia*, *Acinetobacter*, *Reyranella*, *Undibacterium*, *Bryobacter*, *Corynebacterium*, *Cutibacterium*, *Psychrobacter*, and *Wolbachia* were investigated among other relatives. The analysis showed the presence of only one bacterial species for the genera *Spiroplasma*, *Ralstonia*, *Reyranella*, *Bryobacter*, and *Wolbachia* and at least two species for the rest of genera in the sand fly saliva (S1–S10 Figs). The maximum likelihood analysis disclosed that the understudy *Spiroplasma* is phylogenetically related to *S*. *citri*, *Ralstonia* to *R*. *pickettii*, *Reyranella* to *R*. *soli*, *Bryobacter* to *Paludibaculum fermentans* and *Wolbachia* to a *Wolbachia* supergroup G isolated from Thomisidae spider (*Diaea circumlita* species). The reconstructed trees demonstrated respectively that 27 and 16 distinct OTUs of *Corynebacterium* and *Acinetobacter* are present in the saliva of the *P*. *papatasi* sand flies. Three distinct taxa for *Undibacterium* and *Psychrobacter* and two for *Cutibacterium* were also found in the saliva.

### Quantification of bacteria load during sand fly salivation

The model used for bacteria quantification displayed the minimum number of bacteria in the body of sand flies and those that are egested during feeding as 41,205 ($\bar{x}$ = 8,241 per sand fly) and 45,763 ($\bar{x}$ = 9,152 per sand fly) cells, respectively. The number of bacteria egested was 1.1 times the number of bacteria in the guts of the sand flies. Neither smear preparation nor OD measurement were successful in quantifying the bacterial load.

## Discussion

In this research, the quantity and quality of bacteria were studied in the saliva of the sand fly *P*. *papatasi*, the main vector of ZCL. Culture-dependent and culture-independent methods identified *E*. *cloacae* complex members and *Spiroplasma* as dominant taxa, respectively (Tables 2 and 3). The model used to quantify bacteria revealed that each sand fly left at least 9,152 different bacterial cells in the feeding substrate. As emphasized in recent investigations, the above-mentioned bacteria not only can be potential pathogens in the vertebrate host but also may cause more serious consequences when accompanied by the *L*. *major* parasite [18,20].

It is thought that all aspects of leishmaniasis, as one of the most important tropical/subtropical diseases of the world, have been thoroughly investigated. However, besides the main components of the disease (vector, pathogen, reservoir, and host) and their interaction, other factors are involved in the outcome of leishmaniasis, many of which are unknown or little known [20]. In this regard, the role of bacteria—especially those transmitted during vector bites—in the pathogenesis of vector-borne pathogens has largely been overlooked. In line with the studies by Dey et al. and Amni et al., the results of our study identified bacteria as an important part of the life cycle of leishmaniasis (Tables 2 and 3). Bacteria found in an infectious bite can be effective in the wound formation and worsening of lesions [18], as well as may role play in the continuation of the infection and even recovery of the disease [20]. Owing to the fact that the microbiota of sand fly vectors are affected by intrinsic and extrinsic factors, including the microclimate of the location [46,47], wounds that do not heal [48] or in which the symptoms progress or lesions spread [1,49,50] can be attributed to the variation in microbiota of the sand flies.

Forced saliva assay is, by definition, forcing the insect to secrete saliva with the purpose of basic and practical-field experiments (Fig 1). The method are typically used to measure the mosquitoes competence in arboviral diseases [28,51–54], to assess viral load in vector saliva [54] or to characterize the immunomodulatory properties of salivary proteins from different mosquito species [27]. In case of malaria, the forced salivation method is used to estimate extrinsic incubation period in individual mosquitoes and study *Plasmodium*-*Anopheles* interactions [55], It is also used in monitoring and control programs as a rapid detection method for the presence of infectious mosquitoes capable of transmitting malaria [26]. In more limited cases, this technique can be used to understand the interactions between pathogens in co-infections, as reported in previous studies [56–58]. To the best of our knowledge, this is the first study conducted using forced salivation assay in sand flies to investigate bacteria released during salivation, which can help discriminate proven vectors of diseases by identifying *Leishmania* metacyclic parasites and also viruses in their infectious saliva.

In this study, the average success rate of forced salivation was found to be about 44% (Table 1), which seems to be a low success rate. The duration of starvation before testing the sand flies had a direct effect on their salivation success rate. In the current study, we used field-caught specimens, which presumably be more successful with insectary populations. However, the results of the present study are very valuable from the point of view that the sand flies used were from the ZCL hyperendemic focus of Isfahan Province, where *L*. *major* is being transmitted.

The most common method of measuring the abundance of microbes of different niches is to gather DNA samples and sequence a specific gene such as the *16S rRNA* as a “barcode gene” from those samples [59]. Considering several assumptions, it was possible to estimate the frequency of microbes in *P*. *papatasi* saliva based on 16S Illumina sequencing and 16S genomic copy number (Table 3). The applied method has been shown to be valid in estimating microbial communities in numerous habitats [60–64]. The copy number of *16S rRNA* gene can vary from 1 to more than 15 [65], and large copy number variation can cause bias in the estimation of relative abundance of cells and incorrect qualitative interpretations [59]. Therefore, in this study, the average number of copies for each bacterial genus was considered to minimize this error (Table 3). Also, in this study, the V1–V5 regions of the *16S rRNA* gene, showing the least intragenomic heterogeneity in bacteria [66], were targeted, which is another strength of the study.

Sanger sequencing and NGS methods respectively identified *E*. *cloacae* and *Spiroplasma* as the predominant microbes in *P*. *papatsi* saliva (Tables 2 and 3). *Enterobacter cloacae* is a commensal bacterium in the digestive tract of humans and animals as well as insects of medical importance [67], which its natural circulation has been proven among the ZCL partners in the hyperendemic focus of Isfahan Province [30], and after successful genetic manipulation [68] and evaluation of its stability in sugar bait [69], has passed the preliminary experiments to reduce pathogen transmission in the platform of a paratransgenesis approach. Considering the widespread of bacterium in nature and acting as an opportunistic microbe [67], future studies should also pay attention to the possibility of causing infection by *E*. *cloacae* in the vertebrate host during the bite of the vectors.

Spiroplasmas are of special interest due to their unique morphology, motility, and lifestyle, as well as their economic and medical significance as pathogens of plants, insects, and vertebrates [70,71]. The well-known species are *S*. *poulsonii*, reproductive manipulator of flies and also *S*. *citri* and *S*. *kunkelii*, the plant pathogens [72,73], which their sequences clustered within the *Spiroplasma* clade found in this study. *Spiroplasma* has been detected in several fly, mosquito, and sand fly species [74–77]. A study on sand flies revealed the presence of this bacterium in a quarter of females but not in males, which probably specifies the occurrence of a male-killing phenomenon [77]. Although the possibility of infection of humans and other vertebrates with *Spiroplasma* species in natural conditions is largely unknown, the possibility of transmission of these bacteria by blood-sucking insects is not out of mind.

Sand flies secrete saliva while feeding on both sugar [23] and blood [24] meals. The initial strategy of the current study was to identify saliva bacteria during blood feeding of sand flies; however, due to the refusal of field-caught flies to feed on blood, the study was shifted to a sugar meal. It is thought that the process of saliva secretion during feeding from two types of food sources is different, which its investigation requires a special methodology.

While culture-independent Illumina HiSeq sequencing, compared to Sanger method, significantly detected more bacterial taxa (Tables 2 and 3), the dominant taxa from the Illumina data were not identified by traditional culture-dependent methods. All the bacteria observed in the sand fly saliva were also found in the gut (Table 3; Fig 3); however, the culture-independent method determined the number of the egested bacteria more than in the intestine. This observation, in fact, highlights the importance of released bacteria in modulating the vertebrate host’s immune system, pathogen establishment, and pathogenesis.

In previous studies, the average infectious dose of *Leishmania* parasite transmitted by the vector to the vertebrate host was determined to be 1,000 parasites for each sand fly [78,79]. However, the co-occurrence of the parasites with intestinal bacteria as sand fly infectious inoculum has relatively been disregarded. The model studied herein not only verified the transmission of bacteria during the bite but also presented their number to be nine times that of infectious parasites in the vector. The numbers obtained here may not be definitive and may change according to the study method and nutritional physiology of the vector. It is also possible that the forced feeding process egestes more bacteria than normal which should wait for more details in future studies. Moreover, studies on multiple *Leishmania*-sand fly-host combinations have uncovered that pre-exposure of hosts to sand fly bites confers significant protection against *Leishmania* infection [80]. In the distant years, this pre-immunity was attributed to at least 20 diverse salivary proteins with anti-hemostatic and anti-inflammatory properties [24], but studies in recent years have emphasized the role of bacteria transmitted along with saliva [18,20].

Modulation of the host’s immune system may be true for any bacteria identified in the current study. However, it is likely to be more significant for *Acinetobacter* and *Corynebacterium*, which were found with high phylogenetic diversity in saliva (S3 and S7 Figs), and whose immunomodulatory abilities have been previously reported [81,82].

## Conclusion

The absence of a vaccine and treatment failure in leishmaniasis provides many motivations to better understand the factors cause the inflammatory response. The vector gut microbiota, as a major player, can act an important role in modulating the host immune responses for the establishment and spread of a pathogen and even recovery from the disease. The present study determined the type and number of bacteria left in the feeding substrate during the forced salivation of *P*. *papatasi* sand flies caught from the hyperendemic focus of ZCL in Isfahan Province. With the quantification of metagenomic traits, the possibility of exploiting bacteria in the explanation of the infectious inoculum of the sand fly vector became possible. Collectively, the findings of this study can improve our insight into measuring the effect of vector-derived bacteria on the improvement or deterioration of leishmaniasis.

## Supporting information

S1 Fig Maximum likelihood tree inferred from 413–431 bp of the *16S rRNA* gene sequences showing the position of *Spiroplasma* sp. obtained in this study (labeled by a solid diamond symbol) among 38 other *Spiroplasma* spp. reported in the literature. The sequences of *Phytoplasma asteris* (MW661163) and *Anaeroplasma varium* (NR 044663) were set as outgroups. The numbers at the branch points are bootstrap values based on 500 replicates and those lower than 50% were not shown. The bar indicates substitutions per site.
(TIF)

S2 Fig Maximum likelihood tree inferred from 429 bp of the *16S rRNA* gene sequences showing the position of *Ralstonia* sp. obtained in this study (labeled by a solid diamond symbol) among six other *Ralstonia* spp. reported in the literature. The sequences of *Burkholderia cepacia* (NR 029209) and *Burkholderia pseudomallei* (NR 043553) were set as outgroups. The numbers at the branch points are bootstrap values based on 500 replicates and those lower than 50% were not shown. The bar indicates substitutions per site.
(TIF)

S3 Fig Maximum likelihood tree inferred from 423–430 bp of the *16S rRNA* gene sequences showing the position of 16 *Acinetobacter* spp. obtained in this study (labeled by a solid diamond symbol) among 10 other *Acinetobacter* spp. reported in the literature. The sequences of *Moraxella bovis* (NR 028668) and *Alkanindiges hongkongensis* (NR 114676) were set as outgroups. The numbers at the branch points are bootstrap values based on 500 replicates and those lower than 50% were not shown. The bar indicates substitutions per site.
(TIF)

S4 Fig Maximum likelihood tree inferred from 404–405 bp of the 16S rRNA gene sequences showing the position of *Reyranella* sp. obtained in this study (labeled by a solid diamond symbol) among five other *Reyranella* spp. reported in the literature. The sequences of *Stella vacuolata* (NR 025583) and *Constrictibacter* antarcticus (NR 112948) were set as outgroups. The numbers at the branch points are bootstrap values based on 500 replicates and those lower than 50% were not shown. The bar indicates substitutions per site.
(TIF)

S5 Fig The position of *Undibacterium* sp. obtained in this study (labeled by a solid diamond symbol) among five other *Undibacterium* spp. reported in the literature. The sequences of *Oxalobacter formigenes* (NR 029188) and *Oxalicibacterium faecigallinarum* (NR 112834) were set as outgroups. The numbers at the branch points are bootstrap values based on 500 replicates and those lower than 50% were not shown. The bar indicates substitutions per site.
(TIF)

S6 Fig Maximum likelihood tree inferred from 429 bp of the *16S rRNA* gene sequences showing the position of *Bryobacter* sp. obtained in this study (labeled by a solid diamond symbol) among five other *Acidobacteria* reported in the literature. The sequences of *Aridibacter kavangonensis* (NR_133698) and *Blastocatella fastidiosa* (NR_118350) were set as outgroups. The numbers at the branch points are bootstrap values based on 500 replicates and those lower than 50% were not shown. The bar indicates substitutions per site.
(TIF)

S7 Fig Maximum likelihood tree inferred from 422 bp of the *16S rRNA* gene sequences showing the position of *Corynebacterium* spp. obtained in this study (labeled by a solid diamond symbol) among other *Corynebacterium* spp. reported in the literature. The sequences of *Rhodococcus rhodochrous* (NR_037023) and *Mycobacterium tuberculosis* (NR_102810) were set as outgroups. The numbers at the branch points are bootstrap values based on 500 replicates and those lower than 50% were not shown. The bar indicates substitutions per site.
(TIF)

S8 Fig Maximum likelihood tree inferred from 431 bp of the *16S rRNA* gene sequences showing the position of *Cutibacterium* spp. obtained in this study (labeled by a solid diamond symbol) among five other *Cutibacterium* spp. reported in the literature. The sequences of *Escherichia coli* (NR_024570) and *Enterobacter cloacae* (NR_102794) were set as outgroups. The numbers at the branch points are bootstrap values based on 500 replicates and those lower than 50% were not shown. The bar indicates substitutions per site.
(TIF)

S9 Fig Maximum likelihood tree inferred from 430 bp of the *16S rRNA* gene sequences showing the position of *Psychrobacter* spp. obtained in this study (labeled by a solid diamond symbol) among four other *Psychrobacter* spp. reported in the literature. The sequences of *Acinetobacter albensis* (NR_145641) and *Paraperlucidibaca baekdonensis* (NR_117543) were set as outgroups. The numbers at the branch points are bootstrap values based on 500 replicates and those lower than 50% were not shown. The bar indicates substitutions per site.
(TIF)

S10 Fig Maximum likelihood tree inferred from 404 bp of the *16S rRNA* gene sequences showing the position of *Wolbachia* strain obtained in this study (labeled by a solid diamond symbol) among other 18 *Wolbachia* supergroups (WSG: A-T, without supergroup H) reported in the literature. The sequences of *Ehrlichia chaffeensis* (NR_074500) and *Anaplasma bovis* (MH255937) were set as outgroups. The numbers at the branch points are bootstrap values based on 500 replicates and those lower than 50% were not shown. The bar indicates substitutions per site.
(TIF)
