# Supplementary figures and images for: Characterization of bacteria expectorated during forced salivation of the Phlebotomus papatasi: A neglected component of sand fly infectious inoculums
Source: PLoS Negl Trop Dis. 2024 May 21;18(5):e0012165. doi: 10.1371/journal.pntd.0012165 (PMC11108182; doi:10.1371/journal.pntd.0012165)

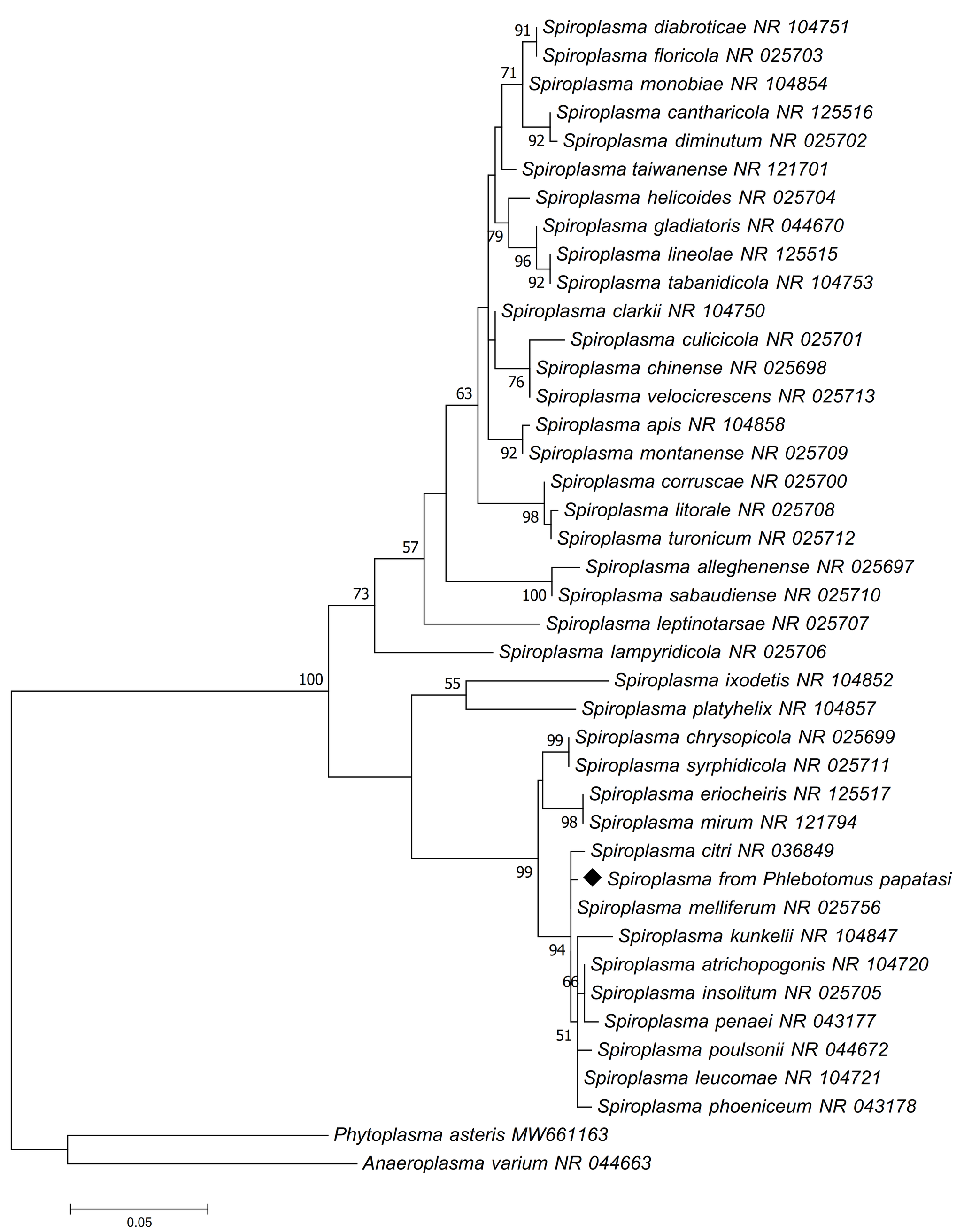

Supplement: S1 Fig — The sequences of Phytoplasma asteris (MW661163) and Anaeroplasma varium (NR 044663) were set as outgroups. The numbers at the branch points are bootstrap values based on 500 replicates and those lower than 50% were not shown. The bar indicates substitutions per site. (TIF) [file pntd.0012165.s001.tif]

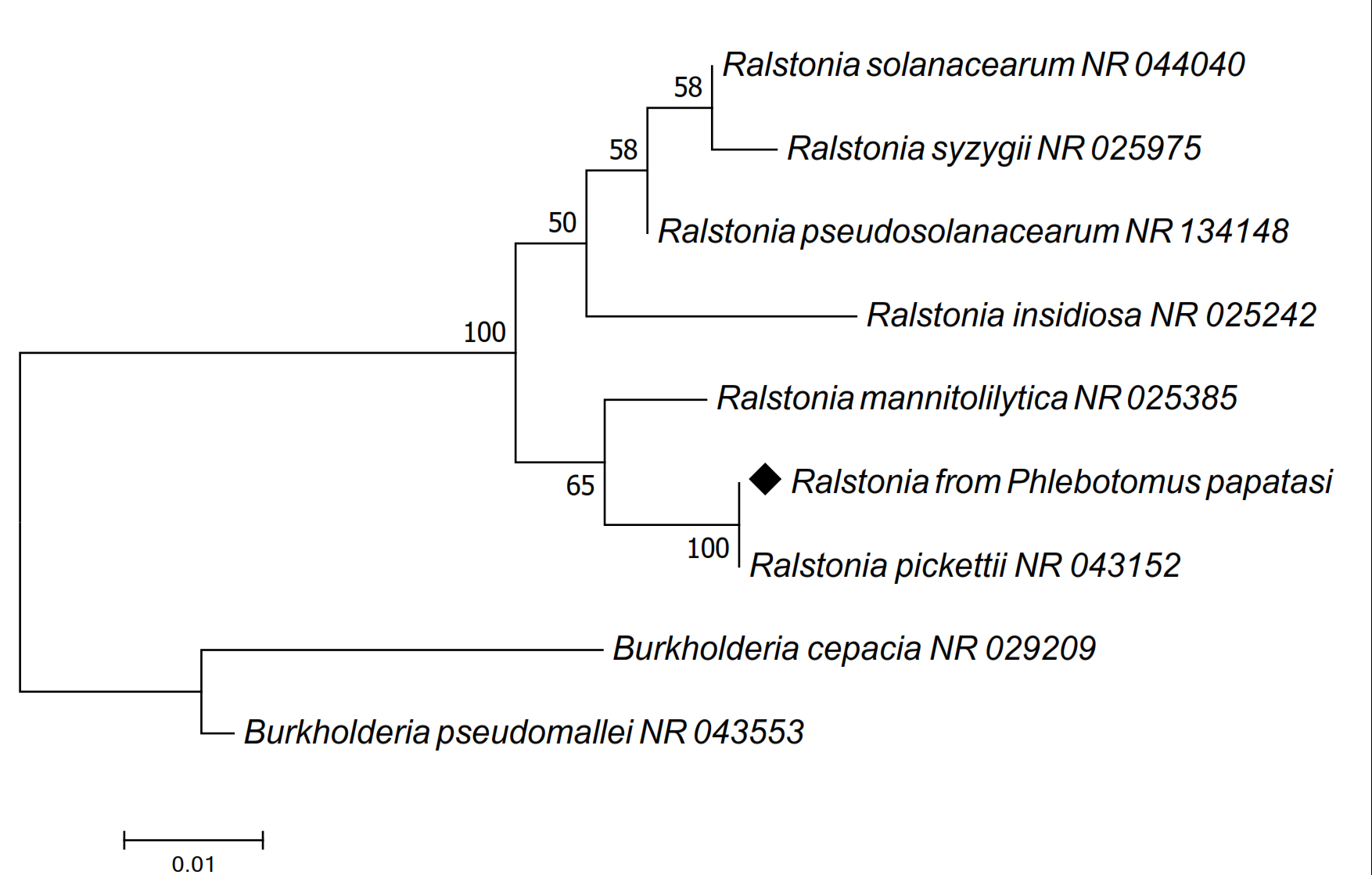

Supplement: S2 Fig — The sequences of Burkholderia cepacia (NR 029209) and Burkholderia pseudomallei (NR 043553) were set as outgroups. The numbers at the branch points are bootstrap values based on 500 replicates and those lower than 50% were not shown. The bar indicates substitutions per site. (TIF) [file pntd.0012165.s002.tif]

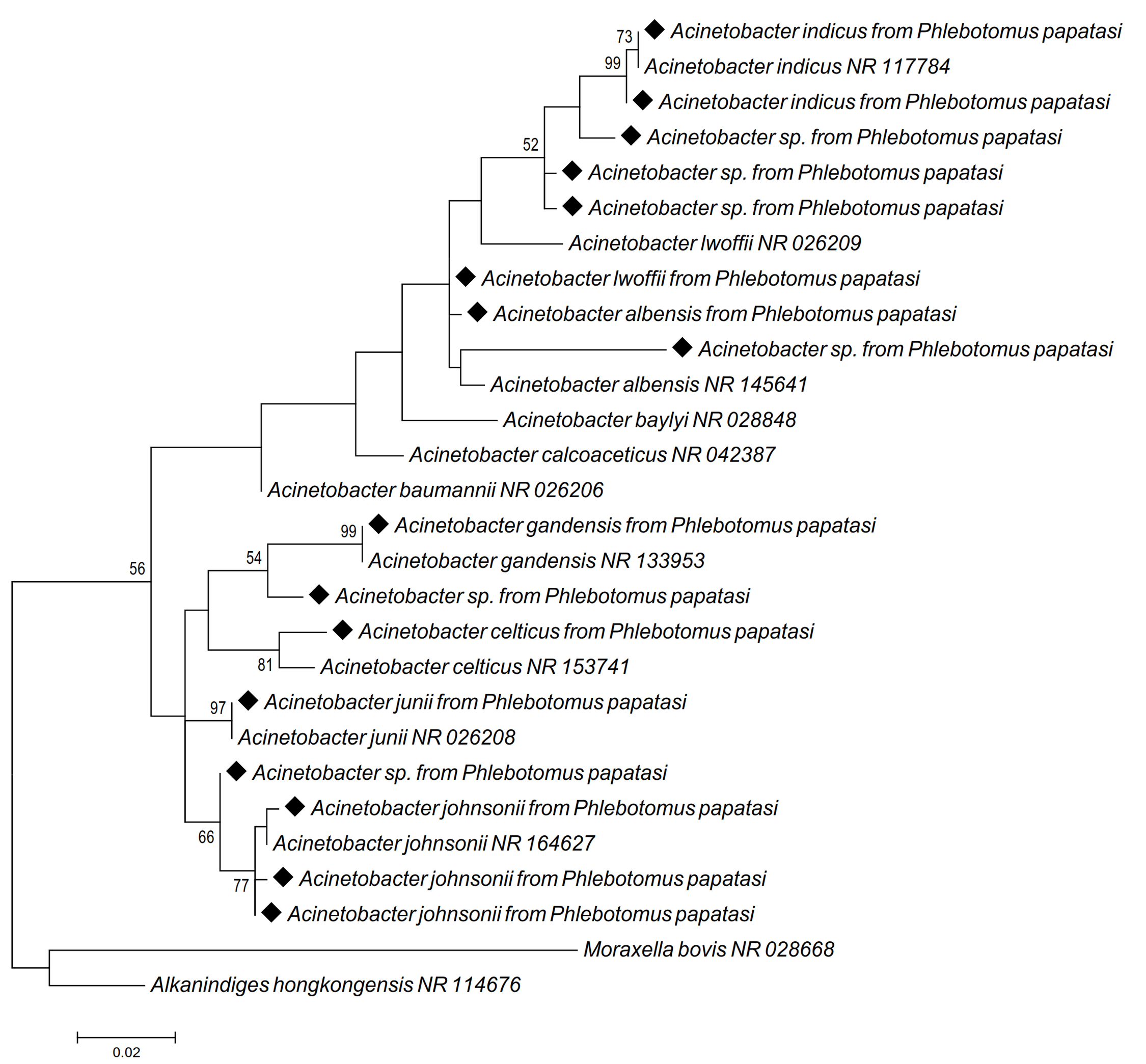

Supplement: S3 Fig — The sequences of Moraxella bovis (NR 028668) and Alkanindiges hongkongensis (NR 114676) were set as outgroups. The numbers at the branch points are bootstrap values based on 500 replicates and those lower than 50% were not shown. The bar indicates substitutions per site. (TIF) [file pntd.0012165.s003.tif]

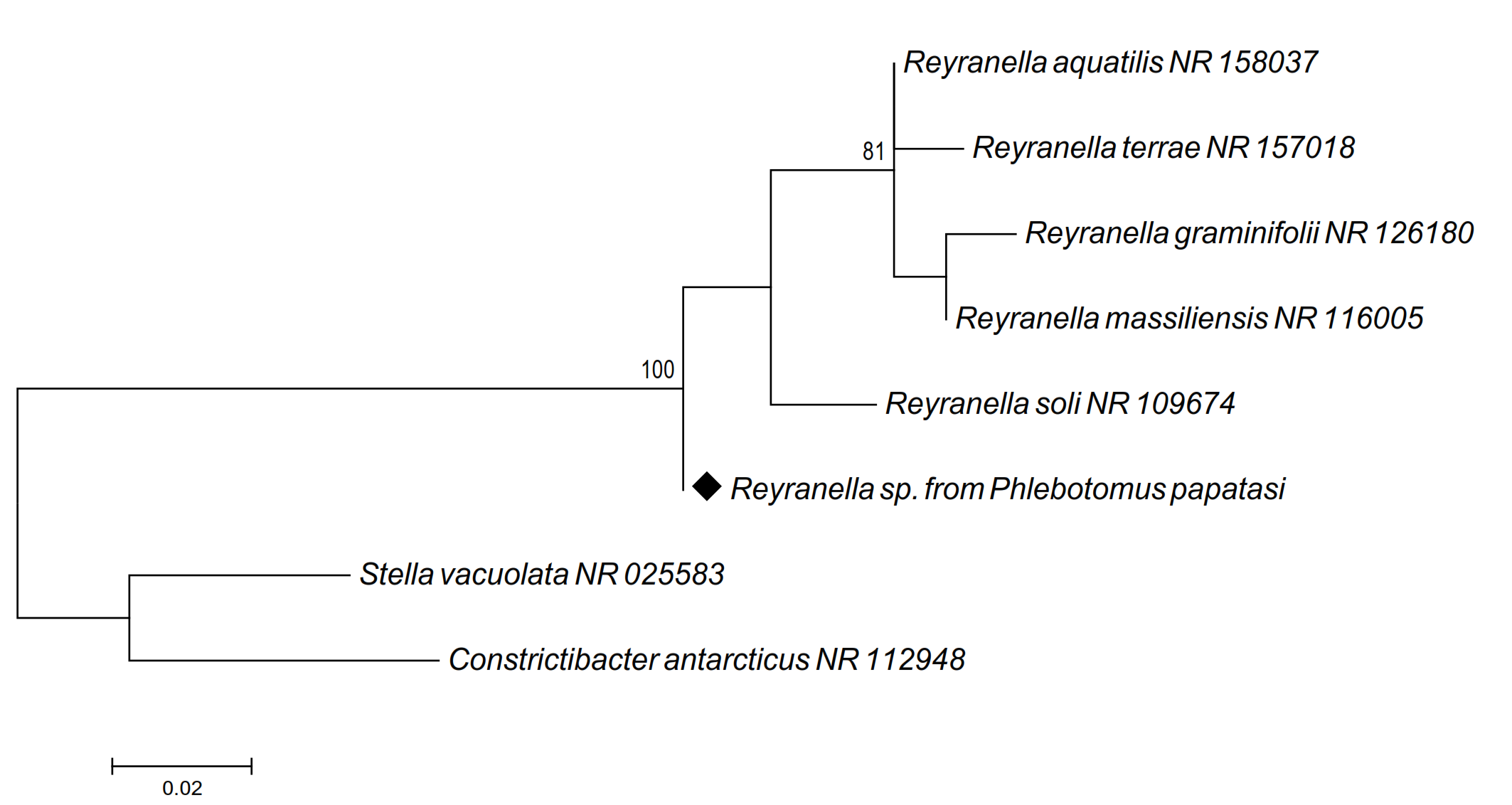

Supplement: S4 Fig — The sequences of Stella vacuolata (NR 025583) and Constrictibacter antarcticus (NR 112948) were set as outgroups. The numbers at the branch points are bootstrap values based on 500 replicates and those lower than 50% were not shown. The bar indicates substitutions per site. (TIF) [file pntd.0012165.s004.tif]

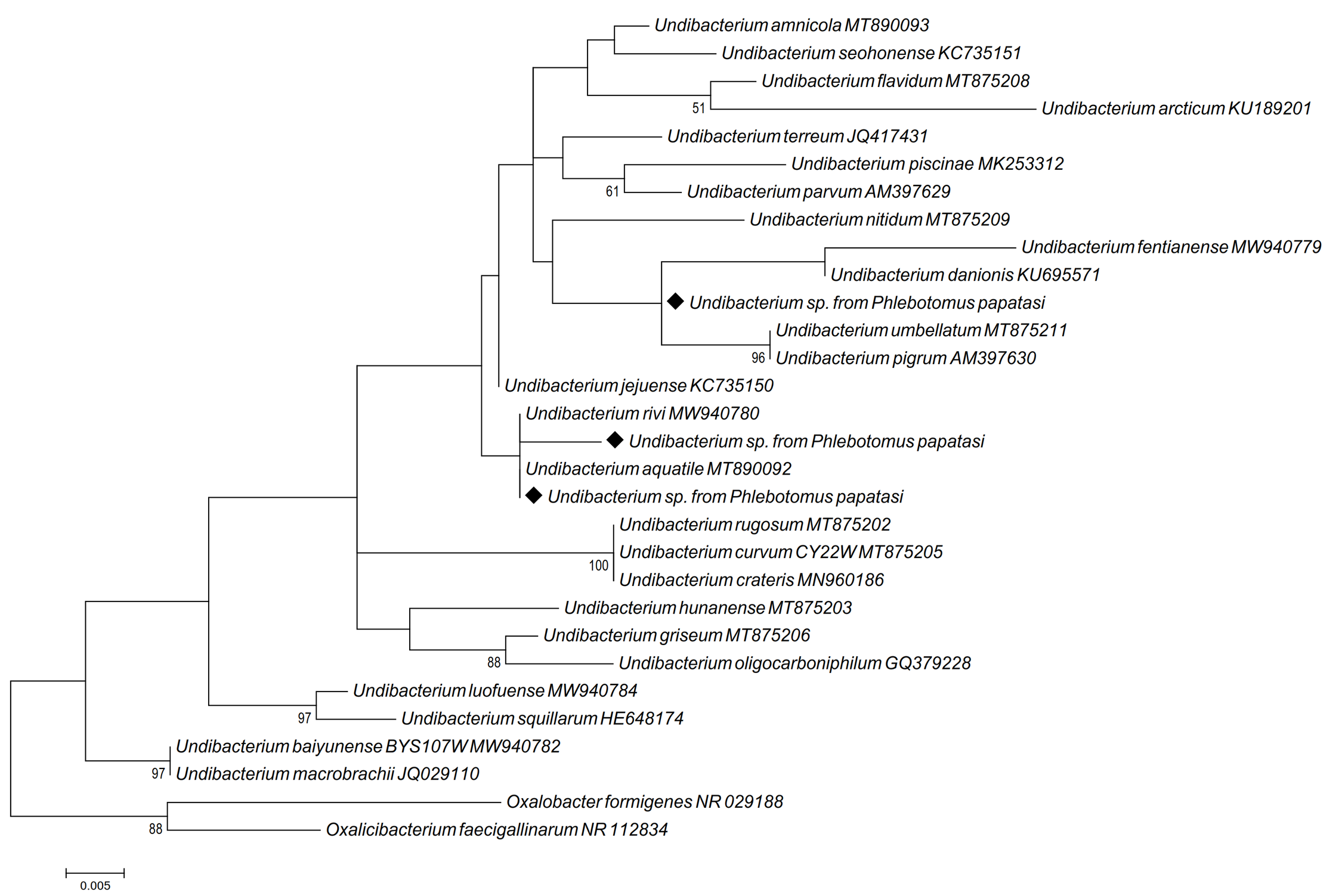

Supplement: S5 Fig — The sequences of Oxalobacter formigenes (NR 029188) and Oxalicibacterium faecigallinarum (NR 112834) were set as outgroups. The numbers at the branch points are bootstrap values based on 500 replicates and those lower than 50% were not shown. The bar indicates substitutions per site. (TIF) [file pntd.0012165.s005.tif]

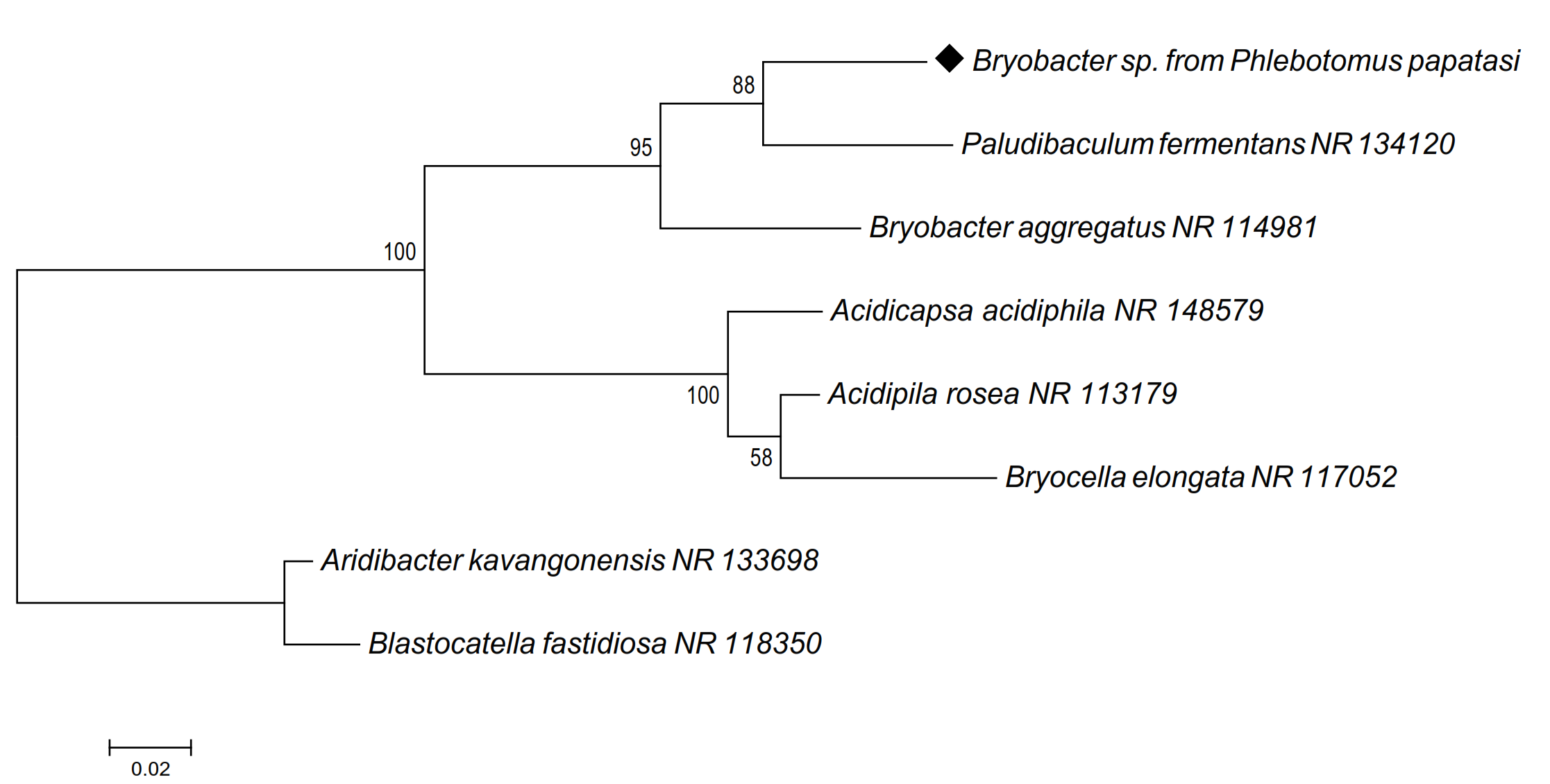

Supplement: S6 Fig — The sequences of Aridibacter kavangonensis (NR_133698) and Blastocatella fastidiosa (NR_118350) were set as outgroups. The numbers at the branch points are bootstrap values based on 500 replicates and those lower than 50% were not shown. The bar indicates substitutions per site. (TIF) [file pntd.0012165.s006.tif]

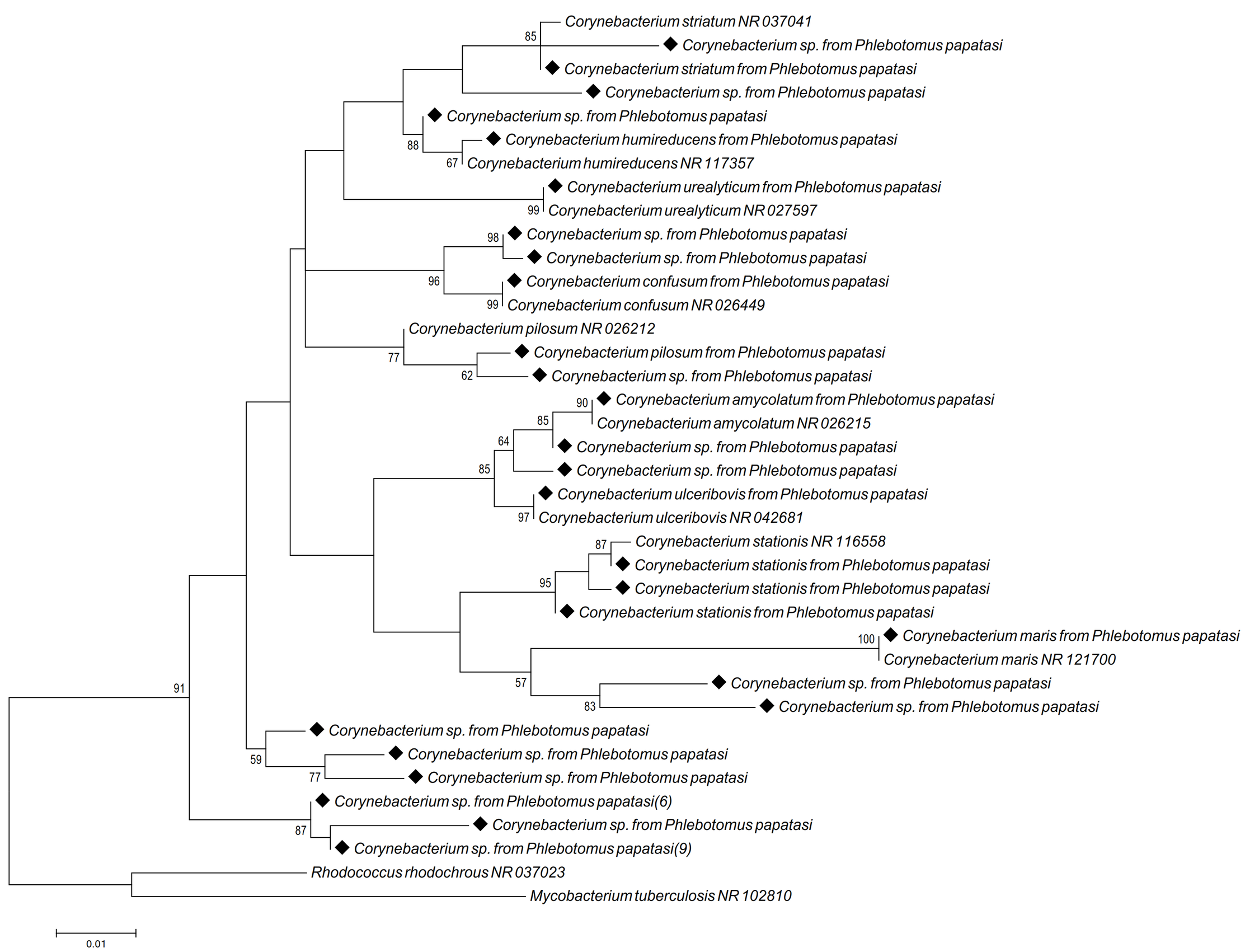

Supplement: S7 Fig — The sequences of Rhodococcus rhodochrous (NR_037023) and Mycobacterium tuberculosis (NR_102810) were set as outgroups. The numbers at the branch points are bootstrap values based on 500 replicates and those lower than 50% were not shown. The bar indicates substitutions per site. (TIF) [file pntd.0012165.s007.tif]

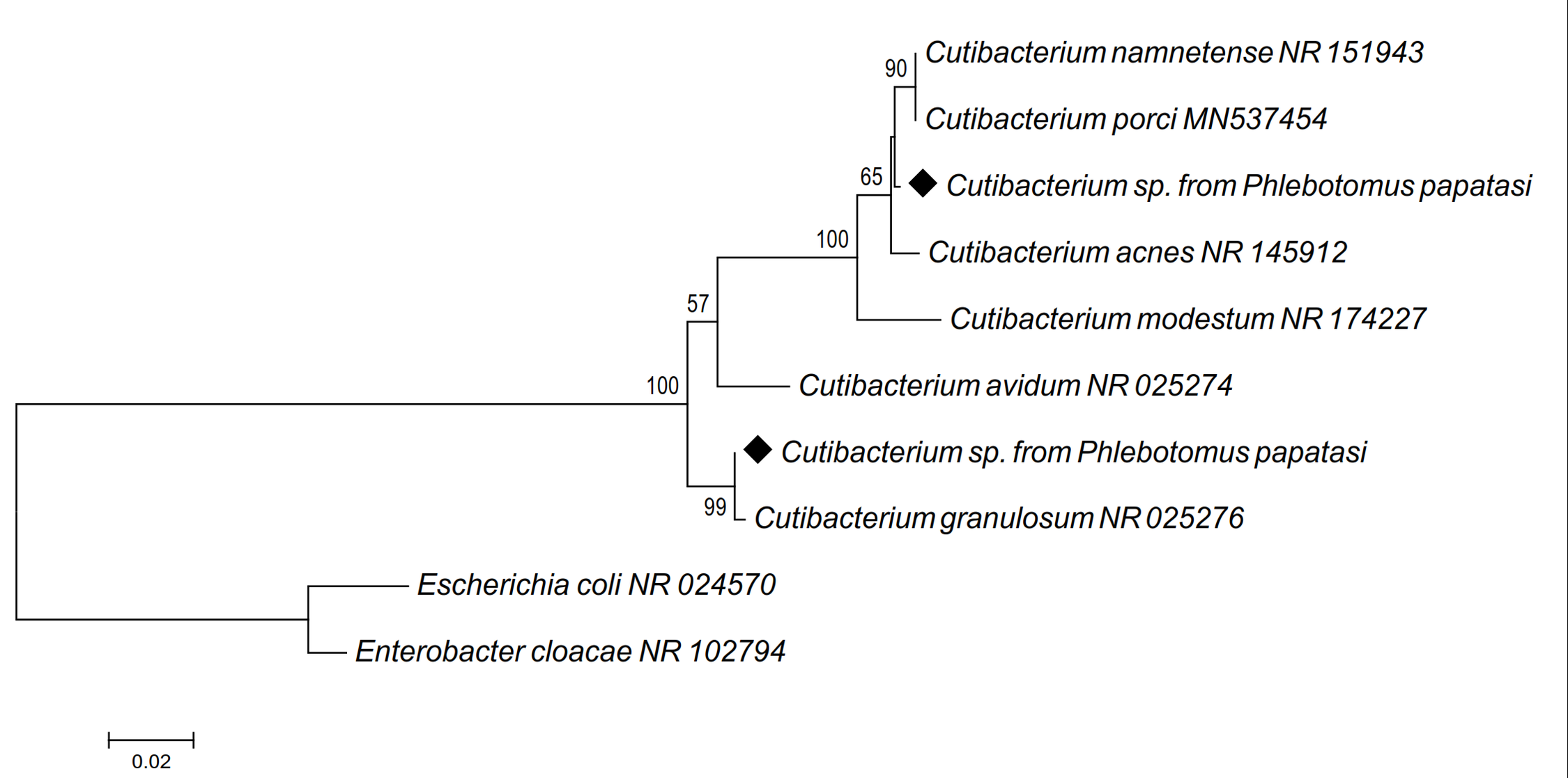

Supplement: S8 Fig — The sequences of Escherichia coli (NR_024570) and Enterobacter cloacae (NR_102794) were set as outgroups. The numbers at the branch points are bootstrap values based on 500 replicates and those lower than 50% were not shown. The bar indicates substitutions per site. (TIF) [file pntd.0012165.s008.tif]

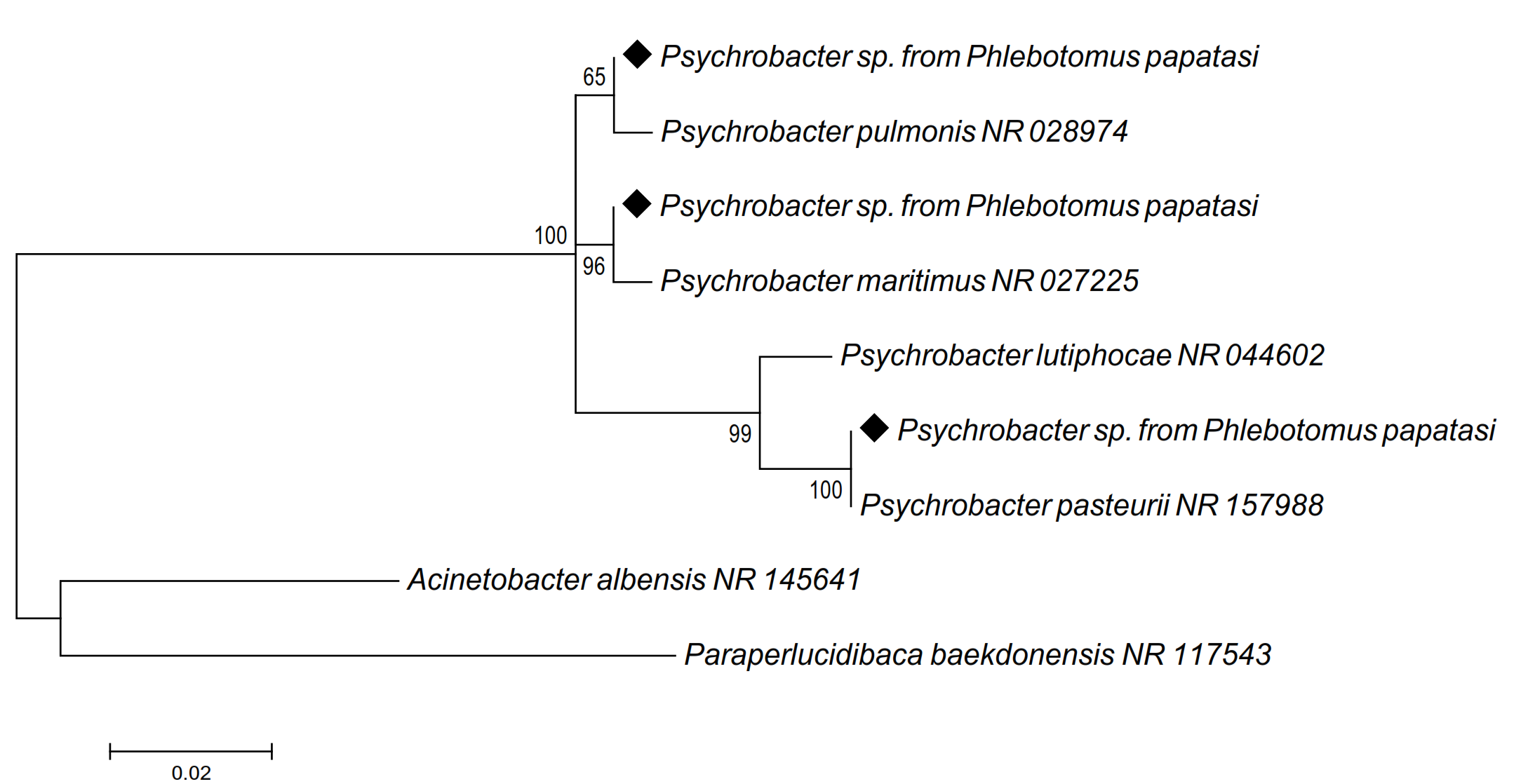

Supplement: S9 Fig — The sequences of Acinetobacter albensis (NR_145641) and Paraperlucidibaca baekdonensis (NR_117543) were set as outgroups. The numbers at the branch points are bootstrap values based on 500 replicates and those lower than 50% were not shown. The bar indicates substitutions per site. (TIF) [file pntd.0012165.s009.tif]

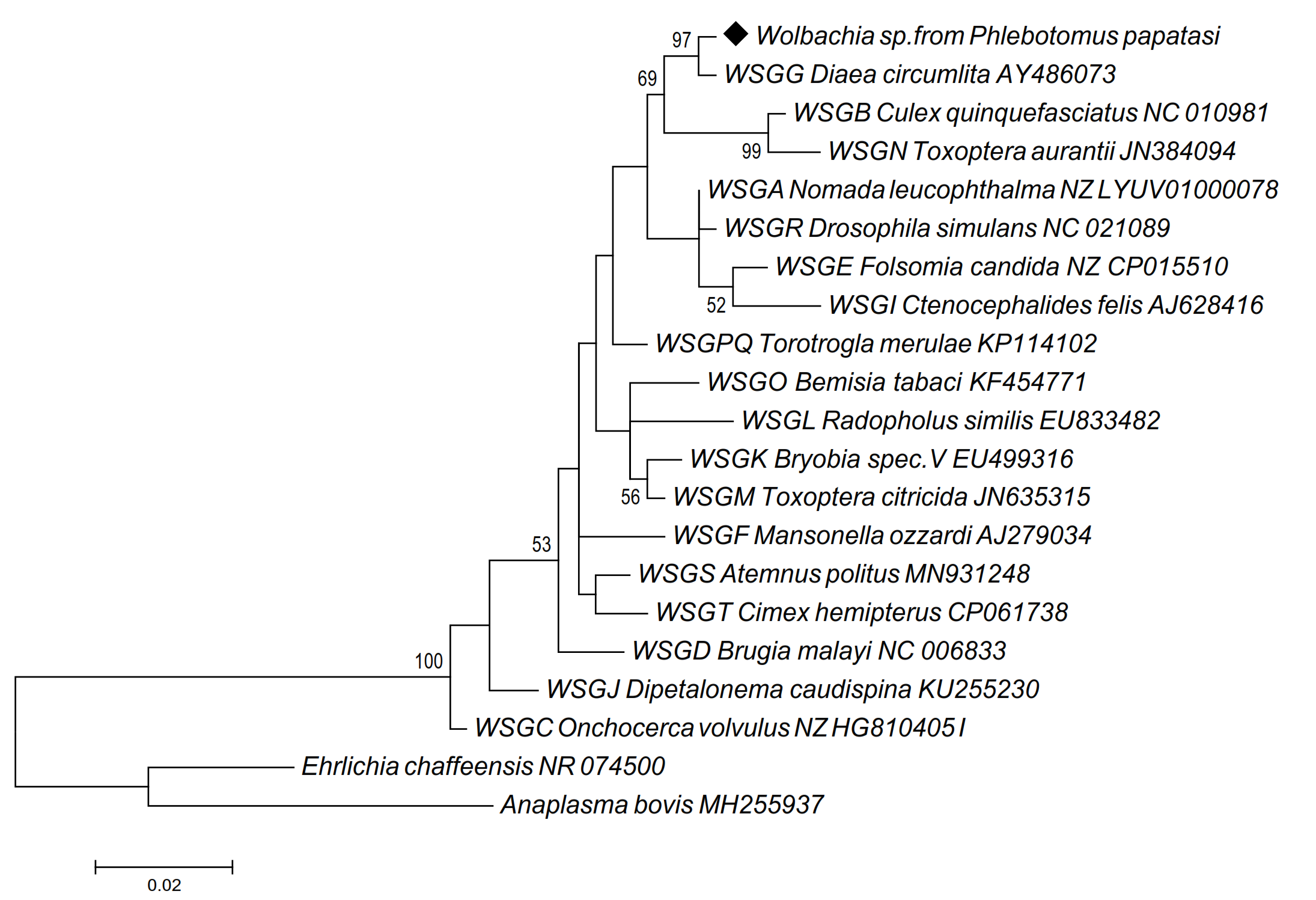

Supplement: S10 Fig — The sequences of Ehrlichia chaffeensis (NR_074500) and Anaplasma bovis (MH255937) were set as outgroups. The numbers at the branch points are bootstrap values based on 500 replicates and those lower than 50% were not shown. The bar indicates substitutions per site. (TIF) [file pntd.0012165.s010.tif]
